# The Relevance and Insights on 1,4-Naphthoquinones as Antimicrobial and Antitumoral Molecules: A Systematic Review

**DOI:** 10.3390/ph16040496

**Published:** 2023-03-27

**Authors:** Gabriela Navarro-Tovar, Sarai Vega-Rodríguez, Elisa Leyva, Silvia Loredo-Carrillo, Denisse de Loera, Lluvia Itzel López-López

**Affiliations:** 1 Facultad de Ciencias Químicas, Universidad Autónoma de San Luis Potosí, San Luís Potosí 78210, Mexico; gabriela.navarro@uaslp.mx (G.N.-T.); sarai.vega@uaslp.mx (S.V.-R.); elisa@uaslp.mx (E.L.); silvia.loredo@uaslp.mx (S.L.-C.); 2 Consejo Nacional de Ciencia y Tecnología (CONACyT), Mexico City 03940, Mexico; 3 Instituto de Investigación de Zonas Desérticas, Universidad Autónoma de San Luis Potosí, San Luís Potosí 78377, Mexico

**Keywords:** naphthoquinones, bioactive secondary metabolites, antitumoral, antimicrobial

## Abstract

Natural product derivatives are essential in searching for compounds with important chemical, biological, and medical applications. Naphthoquinones are secondary metabolites found in plants and are used in traditional medicine to treat diverse human diseases. Considering this, the synthesis of naphthoquinone derivatives has been explored to contain compounds with potential biological activity. It has been reported that the chemical modification of naphthoquinones improves their pharmacological properties by introducing amines, amino acids, furan, pyran, pyrazole, triazole, indole, among other chemical groups. In this systematic review, we summarized the preparation of nitrogen naphthoquinones derivatives and discussed their biological effect associated with redox properties and other mechanisms. Preclinical evaluation of antibacterial and/or antitumoral naphthoquinones derivatives is included because cancer is a worldwide health problem, and there is a lack of effective drugs against multidrug-resistant bacteria. The information presented herein indicates that naphthoquinone derivatives could be considered for further studies to provide drugs efficient in treating cancer and multidrug-resistant bacteria.

## 1. Introduction

Natural products have played a major role in medicinal chemistry for several years. Numerous naturally occurring compounds are an important source of new drugs used to treat some human diseases. The remarkable structural diversity of natural products offers a broad field to discover new compounds with important applications in chemistry, biology, and medicine [1].

Due to the extensive use of pharmaceutical compounds in medicinal treatment, different microorganisms have emerged with enhanced resistance. In addition, many of these compounds have shown strong and adverse secondary effects in humans. As a result, there has been an exponential increase in the efforts to synthesize novel antimicrobial and anticancer agents [2,3,4,5]. Derivatives of natural compounds are an excellent alternative for medicinal treatment since they have been demonstrated to have minor adverse secondary effects compared with synthetic compounds [6].

Based on their structure, quinones have been classified as benzoquinones (containing one ring), naphthoquinones (containing two rings), or anthraquinones (containing three rings). They are extensively distributed in nature and can be found in plants, fungi, algae, and bacteria [7,8]. They constitute a large group of natural and synthetic compounds with important physicochemical and biological properties. Thus, quinones have been applied in several areas, for instance: (1) to develop novel synthetic and heterocyclic derivatives, (2) to be used as molecular probes to study biological interactions, and (3) to synthesize therapeutic agents [7,8,9,10,11,12,13].

**Figure 1:**
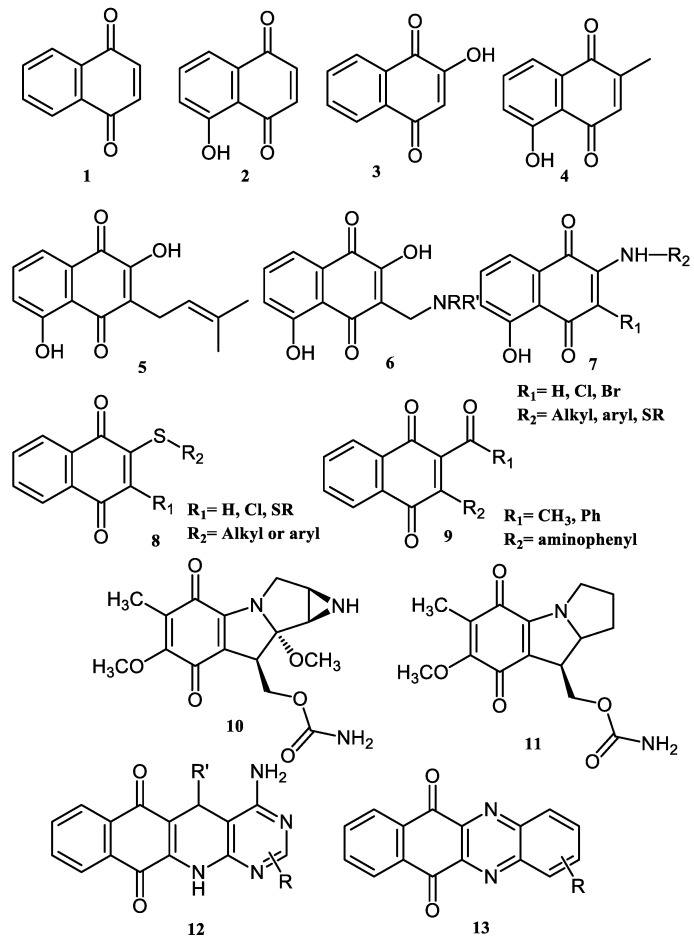
Natural and synthetic NQ derivatives with biological activity.

Naphthoquinones (NQs) contain a naphthalene ring with two carbonyl groups in the 1, 4-positions (**1**, Figure 1). Some NQ derivatives have carbonyl groups in positions 1 and 2. This review focuses only on 1,4-naphthoquinone derivatives since they have been reported extensively as antimicrobial and antitumoral compounds. NQs are found as secondary metabolites in several living species and represent a chemical defense for them [14]. The hydroxyl-1,4 naphthoquinones such as juglone **2**, lawsone **3**, plumbagin **4**, and lapachol **5** are natural NQs isolated from plants extensively used in traditional Indian medicine [15,16,17,18]. In the past decades, the substitution of diverse groups and modifications in the NQs ring have provided many derivatives with enhanced biological activity [19,20,21,22,23,24,25,26,27,28,29,30,31,32]. In this respect, lawsone derivatives, 3-(aminomethyl)-2-hydroxy-1,4-naphthoquinones **6**, or amino naphthoquinone Mannich bases have been synthesized and evaluated as anticancer, antimalarial, antiviral, antifungal, and antibacterial molecules [19,20]; amino **7**, and thioether **8** NQs (Figure 1), as well as the NQs with aromatic amines in carbon two or three, may tune their antifungal and antibacterial activity and anticancer activity, being the case for 2-acetyl-3-aminophenyl-1,4-naphthoquinones **9**, which has been evaluated for in vitro antiproliferative activity against several types of human cancer cells with very promising results [21]. These experimental studies prove the importance of developing different methodologies to prepare novel NQ derivatives.

Many biologically active compounds contain an indole heterocyclic structure [22,23,24]. Examples of this type of compound are mitomycin A **10** and 7-methoxymitosene **11** (Figure 1). Indole is a fundamental structure in medicinal chemistry since it binds to multiple receptors with high affinity. As a result, different compounds containing substituted indole and quinone in their structure show diverse biological activity. The indole quinone subunit is an important framework of the mitomycin family of antitumor agents [22,23,24,25]. Hence, efforts to develop methods to assemble polycyclic compounds containing pyrrole and quinone have been conducted by researchers [26,32]. Among them, pyrido[2,3-*d*]pyrimidine **12** and phenazine **13** ring systems (Figure 1) are principal skeletons among many pharmaceutical scaffolds with applications in synthetic and medicinal chemistry.

To enhance the biological effects induced by NQs, some approaches include incorporating two biologically active groups in the same molecule. For instance, several compounds containing NQ and triazole nuclei in their structure have shown a more potent antibacterial effect than the active groups alone. Therefore, since both groups inhibit bacteria independently, a synergic effect must occur to enhance biological activity [27,28,29,30,31].

This comprehensive review aims to present the obtention of several nitrogen NQ derivatives reported in the literature as potential antibacterial and/or antitumoral agents, along with their preclinical evaluations and the proposed biological mechanisms induced by NQs. The authors used the ISI Web of Knowledge as the principal search tool for this revision. The search for articles containing obtention of nitrogen NQ derivatives included: “1,4-naphthoquinone”, “amino acid”, “Mannich base”, “1,4 Michael addition”, and “triazole” as well as patents and conference papers were excluded, then 75 articles, including reviews and original articles, were considered for Section 1 and Section 2 of this paper, with Section 1 as the introduction and Section 2 discussing the chemical modification of NQ structures. Section 3 shows the pharmaceutical relevance and evidence on the antitumoral and antibacterial effects of NQs in preclinical assays focusing on the antibacterial and antitumor mechanisms proposed in the literature according to evidence at the cellular and molecular levels. Searching with the words “1,4-naphthoquinone”, “ROS”, “antibacterial”, “apoptosis”, “necrosis”, and “docking study”, 17 articles were considered, excluding conference papers and patents. Additionally, three articles were revised to explain oxidative stress. The search was restricted to the past ten years. Lastly, Section 5 discusses the biological evaluations of nitrogen NQ derivatives and aims to collect information on the preclinical evaluations; thus, there was a total of 37 articles in a search including the words “naphthoquinone”, “antiviral”, “antimalarial”, “antitumor”, and “antibacterial” and excluding paper conferences, patents, books, and reviews.

## 2. Chemical Modification of NQs Structures with Nitrogen Groups

**Figure 2:**
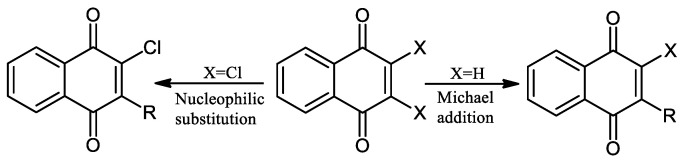
Synthetic strategies to modify the naphthoquinone nuclei.

Due to the biological relevance, the reactions of several nucleophilic atoms with an NQ ring have been extensively studied because the biological activity is related to their redox properties, which can be modulated by the ring substitution [7]. There are two general ways to modify the NQ ring with nucleophilic atoms: (1) Michael 1,4-addition and (2) nucleophilic substitution (Figure 2).

**Figure 3:**
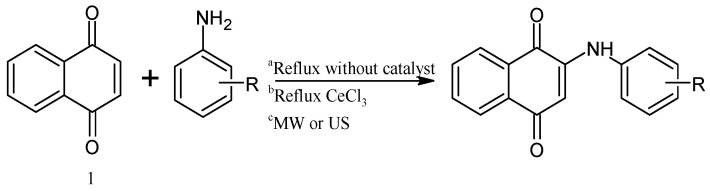
Synthesis of 2-anilino-1,4-naphthoquinone derivatives under different experimental conditions: (a) reflux without catalyst, (b) reflux and CeCl_3_ as catalyst, and (c) microwave (MW) or ultrasound (US) irradiation.

The Michael 1,4-addition inserts nucleophilic atoms as nitrogen or oxygen directly into the NQ. On the other hand, to modify mono- or di-halogenated derivatives, the mechanism is nucleophilic substitution. These reactions could be catalyzed by a Lewis acid and by strong oxidation agents [33,34,35,36]. There are different experimental conditions to perform this reaction, with reflux and no catalyst (a) the reaction takes place in several days, and very low yields are obtained (30–60%). With a Lewis acid catalyst with strong oxidation properties such as CeCl_3_ (b), the reaction takes place in four hours, and high yields of products are obtained (60–90%). Performing the reaction under an alternative source of energy (c), e.g., microwave (MW) and ultrasound (US), results in higher yields (70–95%) of cleaner compounds within minutes (15–50 min) of reaction (Figure 3) [10,37,38,39,40].

**Figure 4:**
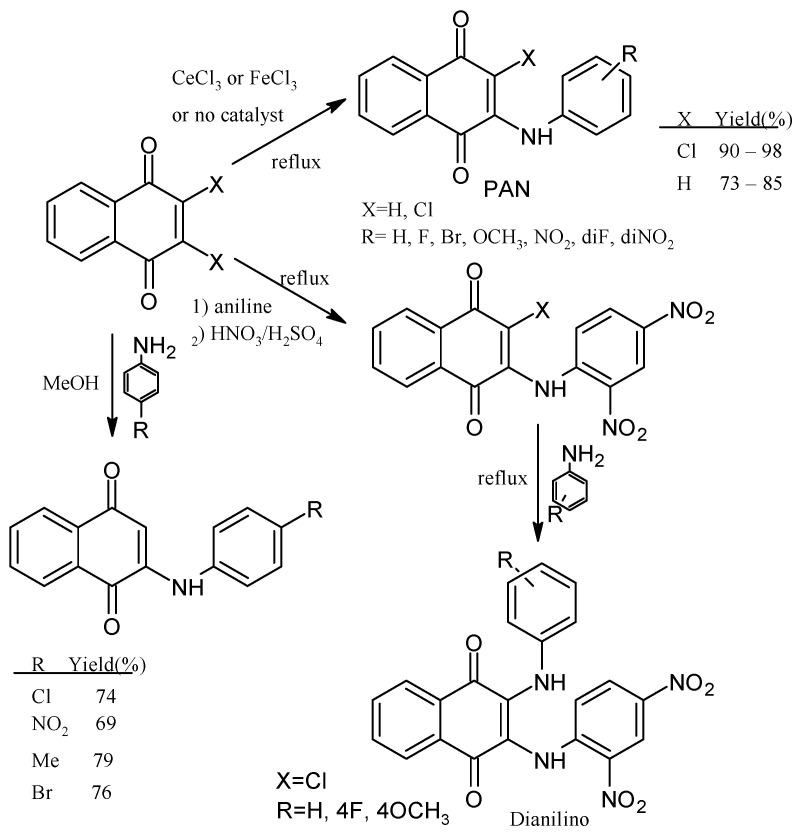
Synthesis of PAN and dianilino derivatives.

Leyva et al., 2017, synthesized anilino (PAN: phenylamino naphthoquinone) and dianilino derivatives. The authors reported a higher yield (>90%) with dichloro-naphthoquinone and no catalyst. The presence of electron-withdrawing groups was necessary to form the dianilino derivative, favoring the second nucleophilic substitution [35]. Moreover, Razaque et al., 2022, prepared four 4-R-PAN by single pot synthesis with no catalyst, in 69–79% yield. Donating groups in the aniline favor the Michael addition [41] (Figure 4).

**Figure 5:**
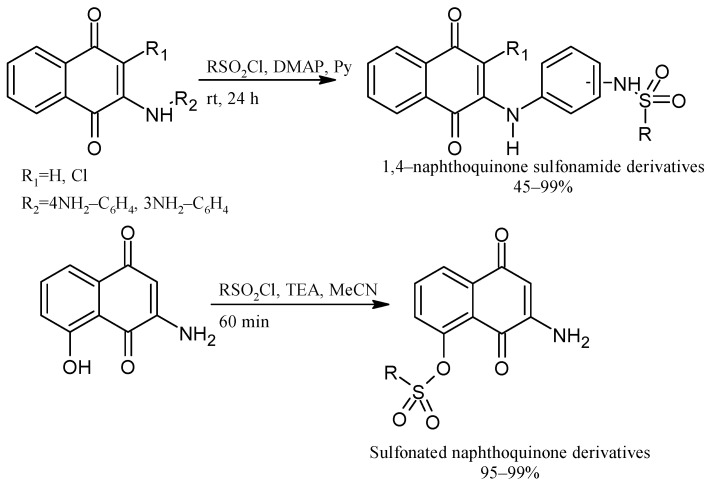
Synthesis of 1,4-naphthoquinone sulfonamide and sulfonate ester derivatives.

**Figure 6:**
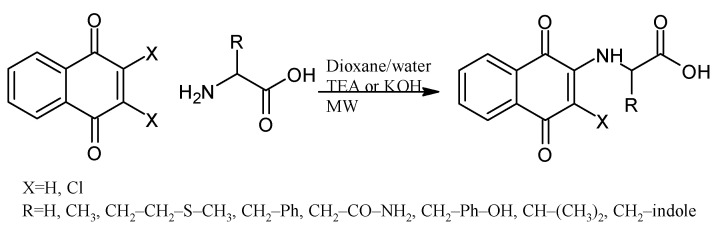
Synthesis of amino acid-naphthoquinone derivatives by Michael 1,4-addition.

Some PANs have been used as starting materials to prepare novel 1,4-naphthoquinone sulfonamide and sulfonate ester derivatives in good yield, as shown in Figure 5 [42]. Rivera et al., 2019, and Araujo et al., 2020, described the modification by Michael 1,4-addition, introducing several amino acids: among them are glycine, alanine, methionine, phenylalanine, asparagine, tyrosine, valine, and tryptophan in good yield (79–95%) (Figure 6) [43,44].

**Figure 7:**
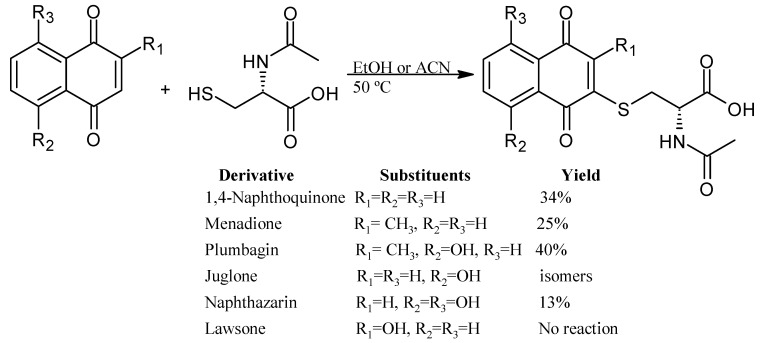
Thia-Michael addition of N-acetyl-L-cysteine to naphthoquinone derivatives.

Micheletti et al., 2022, studied the thia-Michael 1,4-addition of N-acetyl-L-cysteine to the naphthoquinone and the derivatives menadione, plumbagin, juglone, naphthazarin, and lawsone (Figure 7). The reaction with juglone generated two isomers by addition at the 2 and 3 positions. The results show that two hydroxyl groups (naphthazarin) delay the reaction, yielding only 13% after 24 h. On the other hand, the reaction with the derivative with the hydroxyl group in position 2 (lawsone) did not occur, probably due to the tautomeric keto form [45].

**Figure 8:**
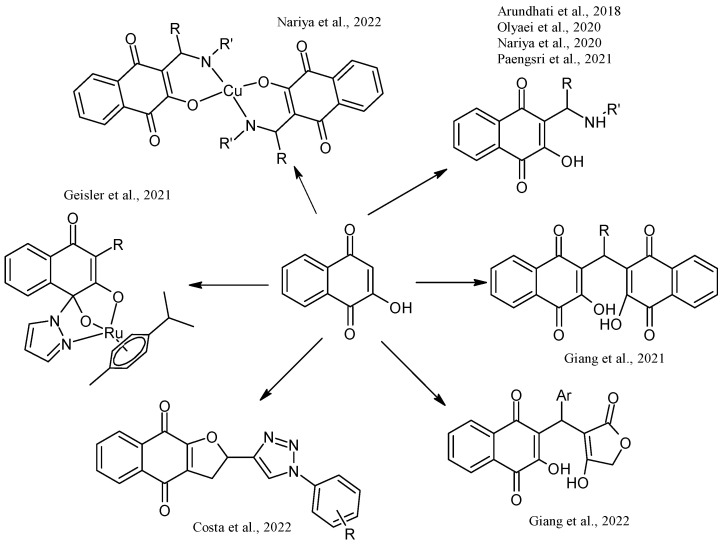
Synthesis of NQ derivatives from lawsone reported by Arundhati et al., 2018 [51], Olyaei et al., 2020 [20], Nariya et al., 2020 [52], Paengsri et al., 2021 [51], Giang et al., 2021 [46], 2022 [47], Costa et al., 2022 [48], Geisler et al., 2021 [49], and Nariya et al., 2022 [53].

Lawsone is used to synthesize Mannich bases that are particularly interesting in medicinal chemistry because of the C-C formation with nitrogen-containing derivatives. The synthesis of 3-(aminomethyl)-2-hydroxy-1,4-naphthoquinones through multicomponent reactions with a non-enolizable aldehyde and primary or secondary amines has been reported [20]. Mannich bases with alkyl, aryl, heteroaryl substituents [46,47,48] and copper and vanadium complexes [49,50] have been reported. Giang et al. used the Mannich reaction to prepare 3,3’-(arylmethylene)bis(2-hydroxynaphthalene-1,4-diones) and 2-hydroxy-3-(arylmethyl)(4-hydroxy-2-oxo-2,5-dihydrofuran-3-yl)-1,4-naphthoquinones under microwave irradiation [46,47]. Furthermore, naphthoquinone-1H-1,2,3-triazoles derivatives have been prepared by oxidative cycloaddition between lawsone and 4-vinyl-1H-1,2,3-triazoles; then, reductive acetylation helped to obtain dihydronaphthofurandiyl diacetates in good yields [48] (Figure 8).

**Figure 9:**
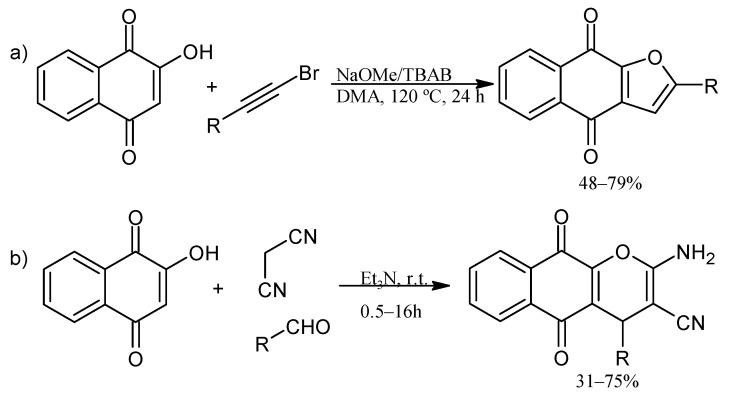
Synthesis of furano (**a**) and pyrano (**b**) NQ derivatives.

**Figure 10:**
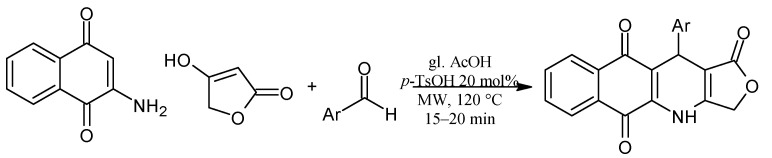
Podophyllotoxin-naphthoquinone derivatives.

In addition, lawsone has been used to prepare naphtho[2,3-b]furan-4,9-dione derivatives via transition metal-free tandem formal [3+2] by a base-promoted alkynylation and subsequent intramolecular addition reaction with aryl bromoacetylenes [50]. The synthesis of pyranonaphthoquinone derivatives has been reported in a multi-component one-pot reaction by a Knoevenagel reaction of malononitrile and aryl aldehydes followed by Michael addition of lawsone (Figure 9) [54]. Moreover, novel podophyllotoxin-naphthoquinone derivatives using microwave-assisted three-component reactions have been reported. The reaction mechanism described a Knoevenagel condensation of 2-amino-1,4-naphthoquinone with aromatic aldehydes, followed by Michael addition, cyclization, and dehydration sequence (Figure 10) [55].

**Figure 11:**
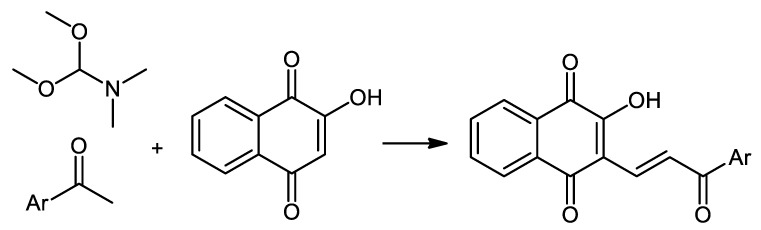
Synthesis of NQ-based chalcone hybrids.

Some interesting molecules are chalcones, which possess various pharmacological properties and have been synthesized via microwave-assisted one-pot three-component reaction (Figure 11) [56].

**Figure 12:**
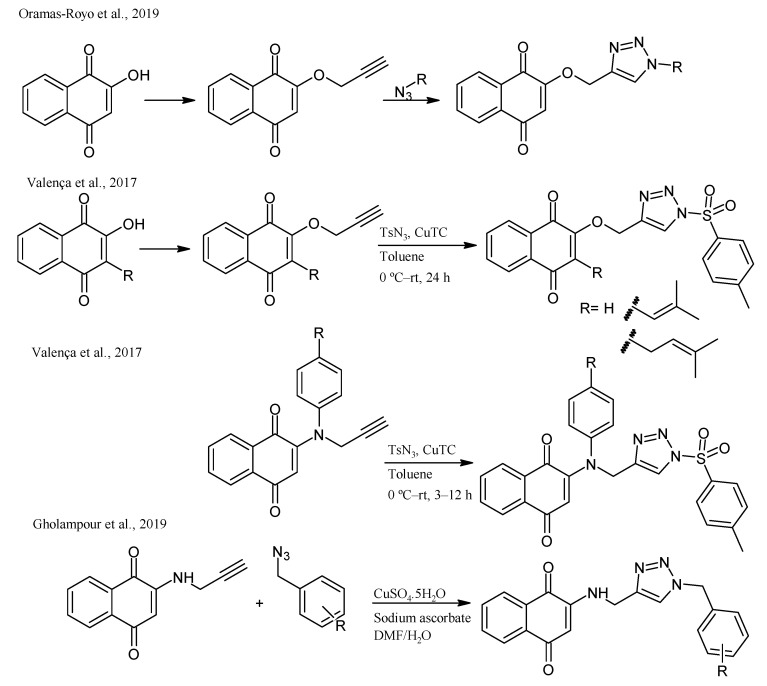
Synthesis of 1,2,3-triazole-naphthoquinones derivatives reported by Oramas-Royo et al., 2019 [57], Valença et al., 2017 [58], and Gholampour et al., 2019 [59].

Moreover, 1,2,3-triazole and its derivatives have been reported as antimicrobial, anti-allergic, analgesic, anti-HIV, anti-inflammatory, anticancer, antimalarial, and antituberculosis agents. Hence, the synthesis of triazole-naphthoquinones derivatives has been studied. O-propargyl-naphthoquinone has been prepared in good yields from lawsone and propargyl bromide. It generates 1,2,3-triazole-1,4-naphthoquinone conjugates in moderate to good yields via click chemistry with alkyl and aryl azides [57]. Valença et al., 2017, synthesized naphthoquinone-based N-sulfonyl-1,2,3-triazoles from lawsone, lapachol, and nor-lapachol through propargylation followed by CuAAC. In addition, aminonaphthoquinones containing 1,2,3-triazoles and sulfonyl triazoles were synthesized [58,59] (Figure 12).

**Figure 13:**
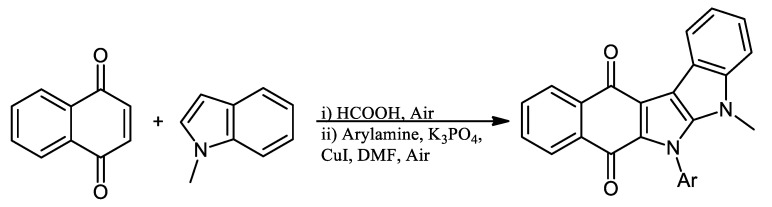
One-pot synthesis of indole-fused NQ derivatives.

Recently, Kumari et al., 2022, reported the indole-fused nitrogen heterocycles by two-step methodology, directly modifying the NQ ring. This compound can be used as solid-state fluorescence material (Figure 13) [60].

The nucleophilic substitution of 2,3-dichloro-1,4-naphthoquinone is a synthetic strategy for introducing nitrogen, oxygen, carbon, sulfur, and selenium nucleophiles at C2 and C3 positions [61].

**Figure 14:**
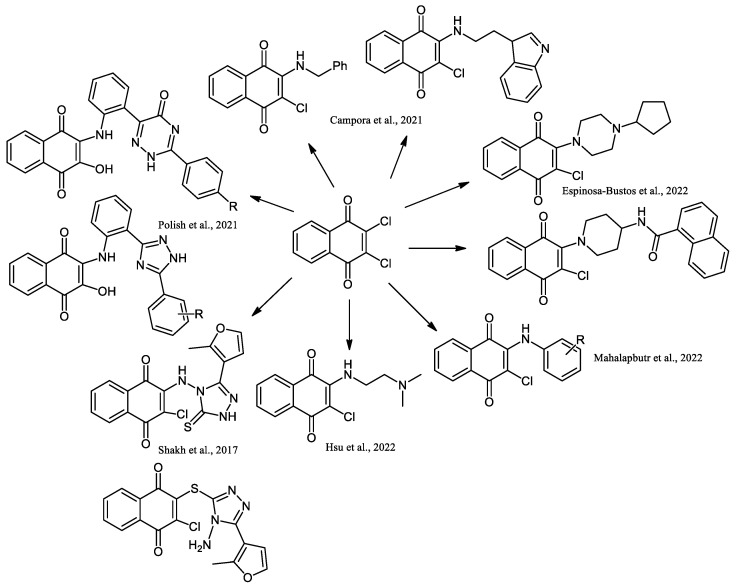
Synthesis of 1,2,4-triazine and 1,2,4-triazole-containing derivatives of 2,3-dichloro-1,4-naphthoquinone reported by Campora et al., 2021 [62], Espinosa-Bustos et al., 2022 [67], Mahalapbutr et al., 2022 [63], Hsu et al., 2022 [64], Shakh et al., 2017 [65], and Polish et al., 2021 [66].

Furthermore, anilino-1,4-naphthoquinone derivatives are compounds with a particular focus due to several biological properties. In the last years, several derivatives have been reported. Campora et al. (2021) reported the synthesis of NQ derivatives bearing hydrophobic moieties as promising candidates for Alzheimer’s disease therapy [62]. Mahalapbutr et al. (2022) presented a set of anilino-1,4-napthoquinone derivatives as potential epidermal growth factor receptor (EGFR) inhibitors targeted for anticancer drug development [63]. The 3-chloro-2-(N,N-dimethylaminoethylamino)-l,4-naphthoquinone, named PPE8, was synthesized by nucleophilic substitution of 2,3-dichloro-1,4-naphthoquinone in benzene with N,N-dimethylenediamine to 93% yield, and the biological studies indicate that PPE8 could be a potential therapeutic agent in the treatment of angiogenesis-related diseases, including cancer [64]. The synthesis of 1,2,4-triazine, 1,2,4-triazole, and 1,2,4-triazole-2-thione naphthoquinone derivatives has also been described [65,66]. Espinosa-Bustos et al., 2022, prepared amino naphthoquinone derivatives as anti-Chagas agents [67] (Figure 14).

The compound 2,3-dichloro-5-nitro-1,4-naphthoquinone was reacted with nucleophiles such as amines, piperazines, or morpholines to form new regioisomeric amino-naphthoquinone derivatives in the C2 and C3 positions [68]. Introducing 3,4,5-trimethoxyphenyl into naphthoquinone amino, piperidine, and piperazine derivatives improved the inhibition of breast cancer cells [69].

**Figure 15:**
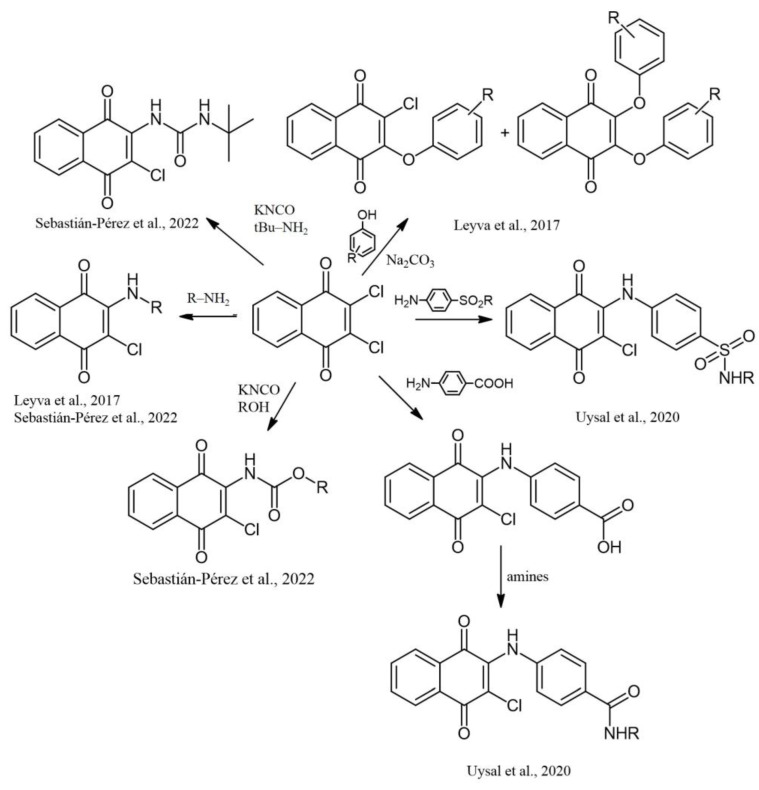
Synthesis of NQ derivatives containing phenoxy, aminobenzensulfonamide, carboxamide, carbamate moiety, an amine group, and urea, reported by Leyva et al., 2017 [35], Uysal et al., 2020 [70], and Sebastián-Pérez et al., 2022 [13].

Synthesis of NQ derivatives containing phenoxy, aminobenzensulfonamide, carboxamide, carbamate moiety, an amine group, and a urea derivative has been reported (Figure 15) [13,35,70].

## 3. Pharmaceutical Relevance and Evidence on the Antitumoral and Antibacterial Effects of NQs in Preclinical Assays

**Figure 16:**
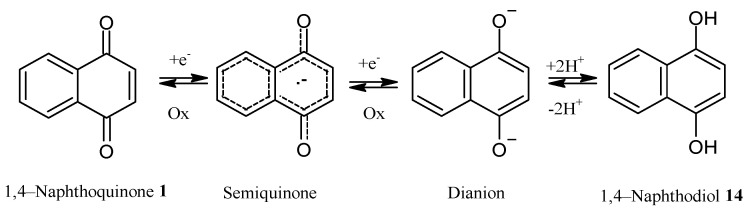
Naphthoquinone oxidation–reduction reactions.

In several cases, the biological activity of NQ derivatives has been explained in terms of their physicochemical property to easily accept one or two electrons to generate a semiquinone or a dianion, respectively (Figure 16). In addition, they can generate a 1,4-naphthodiol **14** by adding protons under solution conditions.

Quinone derivatives can interact with biological structures through several mechanisms. Firstly, quinones have a strong electrophilic character and can form covalent bonds. In solution, these molecules can easily undergo reversible oxidation–reduction reactions. Consequently, they can generate highly reactive oxygen species (ROS) and inhibit electron transport processes and different types of enzymes, such as topoisomerases. Since many quinones have a planar structure, they can function as DNA-intercalating agents [7,10,11,71].

**Figure 17:**
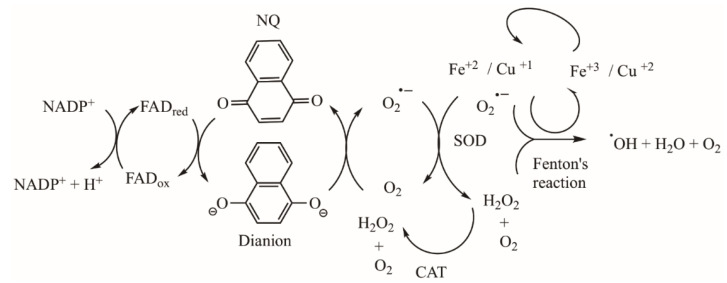
Oxidation and reduction processes induced by NQs at the cellular level.

At the cellular level, the biological activity of NQs has been associated with their redox properties (Figure 17) [10]. Upon acceptance of one or two electrons, the NQ ring easily generates two highly reactive intermediates, namely a semiquinone or a dianion, which are oxidized upon exposure to oxygen and generate several ROS such as superoxide (O2^•–^), hydroxyl radical (^•^OH), and hydrogen peroxide (H_2_O_2_). These latter chemical species can quickly diffuse through membranes, causing cytotoxicity. In addition, ROS quickly induce oxidative stress and apoptosis in cells since they cause damage to biomolecules, such as DNA, proteins, and lipids.

The redox properties and reactivity of a given NQ can be modified by placing different substituents with electron acceptor or electron donor characters in the structure. Therefore, developing easy, fast, and efficient methods to synthesize novel NQ derivatives to find novel compounds with enhanced and adequate biological activity is essential.

### 3.1. Antitumoral and Antimicrobial Mechanisms of NQs

NQ molecules, either from natural sources or semisynthetic, exhibit antitumoral and antimicrobial effects in several biomodels, where some action mechanisms have been demonstrated, such as ROS imbalance, alteration of mitochondrial respiration in tumor cells and bacteria, DNA damage (by alkylation or intercalation), inhibition of topoisomerase II enzyme, among others.

#### REDOX Imbalance (ROS), Alteration of Mitochondrial Respiration, and Other Mechanisms Induced by NQs in Tumor Cells

**Figure 18:**
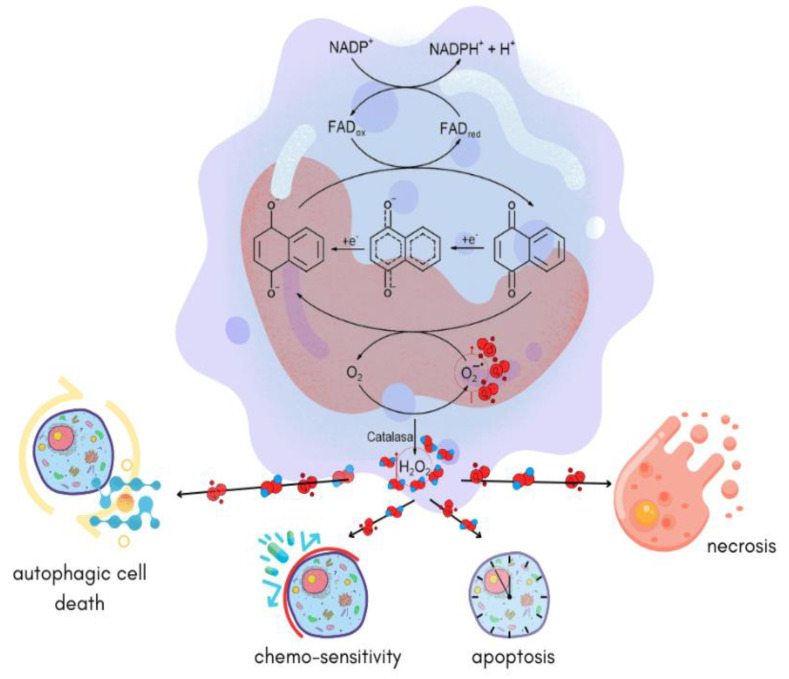
NQs can produce intracellular ROS in tumor cells. The production of ROS can trigger autophagic cell death, increase the entrance and low degradation of antitumoral drugs, and induce apoptosis or necrosis. Figure created based on information from Gambhir et al, 2021 [73].

As previously mentioned, ROS are highly reactive and unstable molecules. Some of them are more reactive since they have an unpaired electron, for example, O_2_^•-^, peroxyl (RO_2_^•^), hydroxyl (HO_2_^•^), hydroperoxyl (HO^•^), and alkoxyl (RO^•^). In living organisms, the primary source of ROS is mitochondrial respiration, where electrons are transferred between protein complexes to produce energy. Some electrons will react with O_2_ to generate O_2_^•-^. The ROS levels are controlled by enzymes, including superoxide dismutase, catalase, glutathione peroxidase, and non-enzymatic antioxidants such as glutathione, vitamins C and E, and any other molecule that could quench free radicals. An imbalance of ROS levels leads to macromolecular damage associated with aging and chronic diseases [72]. However, some physiological conditions trigger the release of ROS by the immune cells to fight against microorganisms and tumor cells. If the production of ROS overpasses the ability of enzymes such as catalase to degrade ROS, then various alterations in the cell signaling lead to cell damage via autophagic cell death, apoptosis, or necrosis (either to microorganisms or tumor cells). In this context, NQs have been extensively reported as a molecule that increases intracellular ROS by producing free electrons in the quinone–semiquinone reaction (Figure 18).

It is well studied that an excess of ROS can alter signaling and gene expression and, consequently, induce tumors; however, ROS are also important in triggering apoptosis. ROS can interact with proteins, such as (1) phosphatases to induce their inhibition, (2) protein kinases (for inhibition or activation) of the Src family, (3) small G proteins, (4) tyrosine kinase receptors of growth factors, (5) and components that induce apoptosis, c-Jun N-terminal kinase (JNK) and p38 kinase (p38MAPK). For example, a small increment in the ROS levels activates the peptidase inhibitor 3-serine/threonine kinase 1 (PI3-K/Akt) pathway; if the ROS levels continue increasing, they trigger p38MAPK-dependent apoptosis [74].

**Figure 19:**
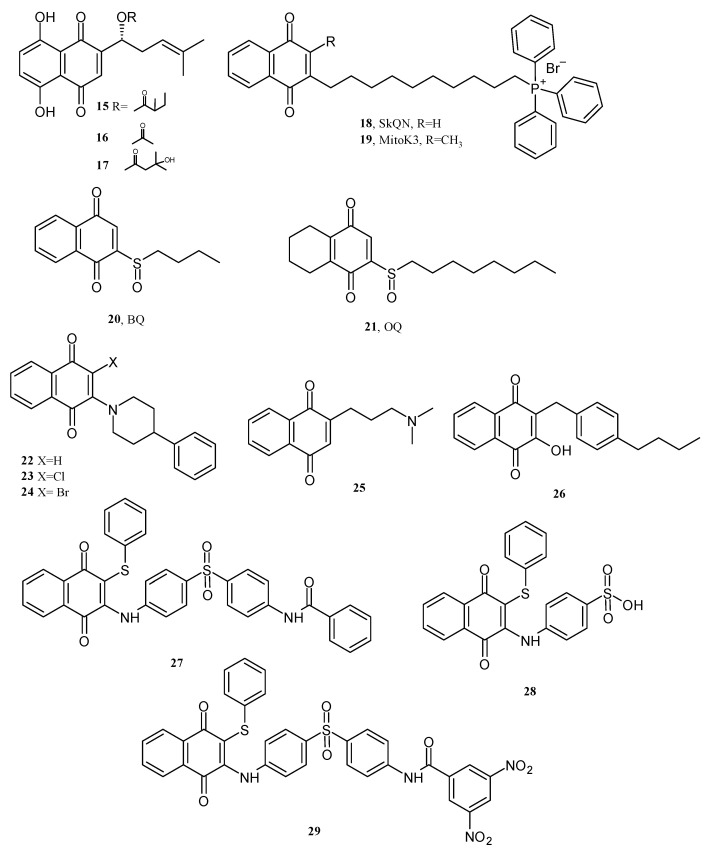
Chemical structures of NQ derivatives reported in antitumoral and antibacterial mechanisms.

As previously mentioned, the NQ structure undergoes transition from a quinone-like structure to semiquinone by one-electron reduction, and in a second step, to hydroquinone. This chemical reaction catalyzes the NADH and O_2_ redox circuit to enhance the intracellular ROS, and several authors have described their impact on cancer cells. For instance, Vukic et al., 2020, evaluated α-methylbutyrylshikonin **15**, acetylshikonin **16**, and β-hydroxyisovalerylshikonin **17** (Figure 19) as prooxidant compounds in the potential treatment of cancer. The evaluation showed an increment in the superoxide anion (O_2_^•-^) and oxidized glutathione levels in human colon cancer cells HCT-116 and MDA-MB-231 cells (regarding non-treated cells) when cultures were treated with 0.1 to 100 µg/mL of NQ derivatives for 24 and 48 h. The authors also reported that levels of antioxidant molecules reduced glutathione (GSH) in the cells treated with any of the NQ derivatives, suggesting that all three derivatives induce oxidative stress [75]. Moreover, Majine et al., 2019, treated C6 glioma cells from rats with natural NQs (10 to 1000 µM): lawsone **3** and plumbagin **4** (Figure 1) and menadione (2-methyl-1,4-naphthoquinone) (Figure 7). The intracellular ROS measured by fluorometry with 2´,7´-dichlorofluorescein diacetate (DCFH-DA) showed a concentration-dependent increment of ROS levels after 3 h of treatment with plumbagin and menadione between 12 and 70% for control (no treatment). However, lawsone treatments reduced the intracellular ROS levels, and these results correlated with the cell viability since plumbagin and menadione (5 to 20 µM) treatments reduced viable cells between 20 and 95%. In comparison, lawsone reduced cell viability by 20 to 40% only when concentrations were from 250 to 1000 µM. The considerable decrease in cell viability was via necrosis. Then, the authors reported that levels of ROS induced by plumbagin and menadione also alter mitochondrial respiration and oxidative phosphorylation and completely uncouple oxidation from phosphorylation (alterations in the electron transport to produce ATP); hence, ATP production decreases, and as a consequence, the necrotic process begins [76].

Those alterations in the mitochondrial function induced by ROS generated by NQs were also observed for modified NQs by Goleva et al., 2020, who reported for two mitochondrial-targeted NQs, [10-(1,4-dioxo-1,4-dihydronaphthalen-2-yl)decyl] triphenylphosphonium bromide (SkQN) **18** and [10-(3-methyl-1,4-dioxo-1,4-dihydronaphthalen-2-yl)decyl] triphenylphosphonium bromide (MitoK3) **19** (Figure 19) that hydrogen peroxide levels and cytotoxicity (IC_50_ 0.3–0.5 µM) for human lung carcinoma cell lines A549 were more significant for the SkQN **18** compound than MitoK3 **19** in mitochondria isolated from rat heart and liver mitochondria. Both compounds (5 to 25 µM) achieved access into the inner mitochondrial membrane, exerted uncoupling activity and hence inhibited ATP production. Additionally, at a concentration of 3 µM, SkQN **18** and MitoK3 **19** induced mitochondrial permeability transition pore opening, an important trigger for apoptosis and necrosis [77].

Wang et al., 2019, studied by flow cytometry using Annexin V-FITC and propidium iodide, the mechanisms involved in the anticancer effect of 2-(butane-1-sulfinyl)-1,4-naphthoquinone (BQ) **20** and 2-(octane-1-sulfinyl)-1,4-naphthoquinone (OQ) **21** on ATTC AGS gastric cancer cells lines. Both BQ and OQ (5 μM at 24 h) induced cell apoptosis (apoptotic rate between 40 and 60%). The same treatment with BQ **20** and QO **21** decreases the levels of Bcl-2, a protein that sequesters proforms of death/driving cysteine proteases (caspases), hence preventing apoptosis [78]. Additionally, BQ **20** and QO **21** increase the levels of biomarkers associated with apoptosis: Bcl-2-associated death promoter (BAD), cleaved-caspase-3 (cle-cas-3), and cleavage of poly(ADPribose) polymerase (cle-PAPR). The authors also reported the depletion in the Akt expression levels, suggesting that BQ **20** and QO **21** induced G2/M phase cell cycle arrest in the AGS cells, hence acting on the apoptotic process [79].

Due to the aromatic structure of NQs, they may also interact with DNA and proteins, altering temporally or permanently the biomolecule’s functions. Espinosa-Bustos et al., 2020, evaluated by cyclic voltammograms the interaction of modified 2-arylpiperidinyl-1,4-naphthoquinone compounds **22**, **23**, **24** (50 µM) (Figure 19) with dsDNA (25 to 100 µL mL^−1^) at 37 °C, pH 7.2 for 45 min. The current peaks related to redox activity for all three compounds decayed as dsDNA concentration increased. Controls showed that free NQ derivatives reduced more easily in the absence of dsDNA. Computational studies evidenced interactions by either covalent or non-covalent interaction of NQ and the DNA structure [80]. On the other hand, the redox NQs activity is also one of the mechanisms affecting tumor cells. Researchers have proposed the coordination of NQs with metals to exert redox activity and interaction with biomolecules. Kosiha et al., 2017, coordinated a 2-((3-(dimethylamino)propyl)amino)-1,4-naphthoquinone **25** (Figure 19) with either Co^2+^, Cu^2+^, Ni^2+^, or Zn^2+^. Solutions of these complexes (0–160 µM) interacting with bovine serum albumin (10 µM) resulted in higher binding constant values (105 M^−1^) than free NQ (103 M^−1^). Additionally, the interaction with CT-DNA (0–50 µM) showed binding interaction as follows: Cu^2+^ > Zn^2+^ > Ni^2+^ > Co^2+^ > free NQ [81].

### 3.2. NQs Alter the ROS Levels and Membrane Integrity and Can Chelate Metals Ions in Bacteria Cells

NQs trigger similar mechanisms in microorganisms, such as bacteria. In the past years, the chemical modification of NQs has been conducted to enhance the selectivity and antibacterial action of NQs. This is the case of Song et al., 2020, report for a lawsone derivative **26** (Figure 19), which can alter the ROS levels and induce cell membrane damage and chelation of intracellular iron ions in a methicillin-resistant *Staphylococcus aureus* (MRSA) model. When MRSA was exposed to **26** (16 μM, 4xMIC, 60 min), propidium iodide (a membrane-impermeable dye) uptake significantly increased, suggesting that the semisynthetic molecule can alter the bacteria cell membrane more effectively than vancomycin and lawsone. The authors linked the cell membrane alterations to intracellular ROS levels increasing (two-fold for nontreated bacteria, 60 min) in MRSA. Moreover, the higher the concentration of **26** in the treatments, the lower the intracellular ion iron levels. The bacteria viability was rescued when an excess of Fe^3+^ was incorporated into the MRSA cultures, suggesting that NQ derivatives could chelate metal ions [82].

Another study considered three NQ derivatives: N-(4-((4-((1,4-dioxo-3-(phenylthio)-1,4-dihydronaphthalen-2-yl)amino)phenyl)sulfonyl)phenyl)benzamide **27**, N-(4-((4-((1,4-dioxo-3-(phenylthio)-1,4-dihydronaphthalen-2-yl)amino)phenyl)sulfonyl)phenyl)-3,5-dinitrobenzamide **28**, and 4-((3-chloro-1,4-dioxo-1,4-dihydronaphthalen-2-yl)amino)benzenesulfonic acid **29** (see Figure 19) for antibacterial tests in *S*. *aureus*, *Listeria monocytogenes*, *Escherichia coli*, *Pseudomonas aeruginosa*, and *Klebsiella pneumoniae*, finding MIC to be between 15.6 and 500 μg/mL. Ravichandiran et al., 2019, also reported that the levels of intracellular ROS determined by DCFH-DA fluorescent dye staining were equal in fluorescence intensity for NQs (15 to 31 μg/mL) in *E*. *coli* to the treatment in the same bacteria with streptomycin (1.9 μg/mL), indicating that antibacterial activity of NQ derivatives is led by oxidative stress caused by ROS [83].

### 3.3. Computational Studies in the Search for NQ Mechanisms against Cancer Cells and Bacteria

To understand the possible biological mechanistic action triggered by different NQ chemical structures, structure–activity relationship (SAR) and molecular docking studies are relevant. Firstly, SAR studies have made it possible to know that the position of substituents is critical for antibacterial activity. In a SAR study, Wellington et al., 2019, reported that the group fluoro in position C3 exhibited better MIC (31.3 μg/mL) against *E*. *coli*, while fluoro in position C4 decreased the antibacterial activity about three times. Removing fluoro groups and adding 3-sulfanylpropanoic acid reduce the MIC by about seven times [84]. Similar evidence presented by Sánchez-Calvo et al., 2016, states that the halogens chloro and bromo in position C2 enhance the MIC (2 and 16 μg/mL, respectively) against *Candida krusei*. The authors also discuss that a single OH in position C5 is essential in antibacterial activity, while methoxylation in C5 and/or C8 is inactive for yeasts [85].

Moreover, NQ reports are frequently accompanied by molecular docking, a computational tool to understand the interaction between NQs and biological ligands. In this sense, molecular docking studies have shown that some NQs, such as juglone **2**, propionyl juglone, and 2-acetyl-8-methoxy-1,4-naphthoquinone (Figure 1), possess inhibitory activity against SARS-CoV-2’s main proteinase since NQ molecules fit into the proteinase through hydrogen bonds with amino acid residues. Jiahua et al., 2021, showed the strongest interaction for 2-acetyl-8-methoxy-1,4-naphthoquinone, which presented hydrogen bonding interactions with His41, Gly143, and Glu166, which explain the highest inhibitory activity against the proteinase [86].

On the other hand, most of the information regarding NQ–biomolecule interactions comes from molecular docking studies. In this sense, Ravichandiran et al., 2019, carried out a molecular docking study to determine if compound **29** (Figure 19) interacts with the *E*. *coli* DmsD protein, which blocks redox proteins from early transport. The interactions have an affinity energy of 2.63 kcal/mol with hydrogen-bonding and π-π stacking forces in the active site of ARG A15, reinforcing the evidence of NQ–protein interactions and ROS imbalance as antibacterial activity and suggesting that NQ derivatives can alter bacteria replication [83].

**Figure 20:**
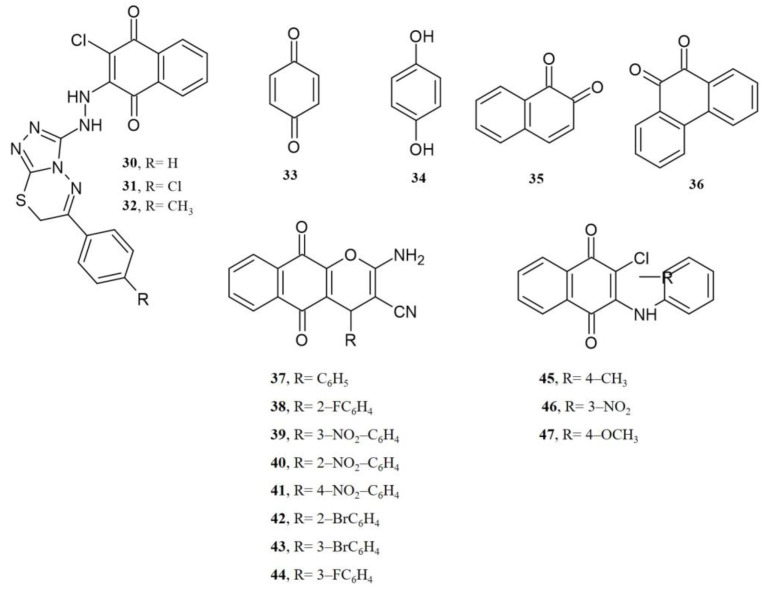
Chemical structures of NQ derivatives reported in computational evaluations.

Thus, it is relevant to know the potential targets in bacteria and cells to increase the efficacy in synthesizing active NQs. Mohamady et al., 2020, designed and synthesized naphthoquinone-hydrazinotriazolothiadiazine analogs based on the molecular docking (HYBRID docking module of OpenEye software) results, where compounds **30**, **31**, and **32** (Figure 20) had a potential interaction with the ATP catalytic binding domain of topoisomerase-II β (Topo-II PDB ID:3QX3). In this study, the hydrazine hydrogen nesting to the triazole ring showed hydrogen bond interactions with the residue ASP479A. The experimental studies in the same report demonstrated that compounds **30**, **31**, and **32** inhibited Topoisomerase IIB (0.55 to 0.64 μM) and induced high cytotoxicity of **31** in HepG2 and MCF-7 cancer cell lines, leading to upregulation of Caspase 3 biomarker, as described in the experiment in [87]. In the same context, Deepak Gurbani et al., 2011, performed experimental studies and molecular docking to understand the mechanism of genotoxicity of 1,4-benzoquinone **33**, hydroquinone **34**, 1,2-naphthoquinone **35** (Figure 20), 1,4-naphthoquinone **1** (Figure 16), and 9,10-phenanthroquinone **36** (Figure 20). The molecular docking of these quinones with the ATPase domain of the human topoisomerase IIα predicted that these compounds interact with Ser-148, Ser-149, Asn-150, and Asn-91 residues through hydrogen bonds. The synergy between experimental and computational studies allowed the authors to elucidate the possible inhibition mechanism of ATPase [88].

Recently, a group of 2-amino-4*H*-naphthopyran-3-carbonitrile NQ derivatives **37**–**44** was evaluated with a similar perspective by Amani et al., 2023. The authors first evaluated the anticancer activity in HCT116 human colon cancer cell lines. Then, those compounds with the highest cytotoxic activity were taken to assess their interactions in a docking study with the human tyrosine kinase CK-2 (a protein involved in cell growth and proliferation). The findings pointed out that NQ derivatives require a planar aromatic region with substitution to form electrostatic interactions with Lys68 and Asp175 residues. A second interaction was revealed as a non-coplanar aromatic region with π–π stacking forces with His160 [89].

Panupong Mahalapbutr et al., 2022, performed a molecular docking study to reveal the interaction of the 2-chloro-3-((4-methyl-phenyl)amino)1,4-naphthoquinone **45**, the 2-chloro-3-((4-nitrophenyl)amino)1,4-naphthoquinone **46**, and the 2-chloro-3-((3-nitrophenyl)amino)1,4-naphthoquinone) **47** with the epidermal growth factor receptor (EGFR) tyrosine kinase where these compounds fit within the ATP of the EGFR by means of van der Waals, H-bonding, π–π, and alkyl interactions, with 2-chloro-3-((4-methyl-phenyl)amino)1,4-naphthoquinone as the compound with significant interactions and the highest EGFR inhibitory activity [63].

Current experimental and computational evidence highlights several NQ derivatives as antitumoral and antibacterial potential drugs, with more than a single mechanism in eukaryotic and prokaryotic cells.

## 4. Biological Evaluations of Nitrogen NQ Derivatives

The activity of a given substituted anilino naphthoquinone has been associated with its redox properties. Depending on the substituents in the aromatic ring, the amino group in the naphthoquinone can modulate its physicochemical properties and modify its biological activity and interactions with biomolecules [33,36,37,38]. The secondary amine, 2-anilino-1,4-naphthoquinone, is a basic structure present in many natural and synthetic compounds. Many of these compounds have shown important biological properties as antibacterial, antifungal, antimalarial, or anticancer agents. Lawsone Mannich bases are 2-hydroxy-3-(aminomethyl)-1,4-naphthoquinone compounds with important biological activities such as antiparasitic, antibacterial, anticancer, and antiviral, which have a particular interest in medicinal chemistry because the Mannich reaction forms a C-C bond with nitrogen-containing derivatives. This section presents molecules with promissory activity, as shown in Table 1 [31,42,43,44,45,90,91,92].

## 5. Conclusions

As shown in this review, the redox properties of NQs produce ROS as superoxide, hydroxyl radical, and hydrogen peroxide; ROS can trigger autophagic cell death, chemo-sensitive, apoptosis, and necrosis. Furthermore, NQ derivatives can induce cell membrane damage, generate intracellular chelation with metals as iron, and NQ derivatives that preserve the planar structure may intercalate with DNA. Thus, pieces of evidence in preclinical assays (mostly in vitro) highlight to NQs as potential antimicrobial and antitumoral drugs. However, to the knowledge of the authors, no clinical evaluations have been conducted, since NQs face some challenges: (1) low solubility and (2) potential cytotoxic effect over healthy eucaryotic cells. It seems that nitrogen NQ derivative compounds could face those disadvantages, and further studies, particularly rigorous toxicological evaluations, can reveal the new generation of NQs for commercial applications.

## Figures and Tables

**Table 1:** Biological evaluations of aniline-, -amino acids- and Manich bases naphthoquinone derivatives.

Biological Evaluations of NQs-Aniline				
Name	Chemical Structure	Potential Application	Evidence from Preclinical Assays	Ref
2-Chloro-3-((2-(piperidin-1-yl)ethyl)amino)naphthalene-1,4-dione	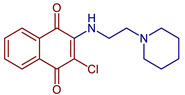 **48**	Antibacterial	MIC of 31.2 µg mL^−1^ against *S. aureus* (209-P) and 15.6 µg mL^−1^ for *M. luteum* (B-917).	[91]
2-((2-Hydroxypropyl)thio)-3-((3-(trifluoromethyl)phenyl)amino)naphthalene-1,4-dione	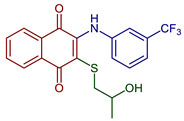 **49**	Antibacterial	MIC of 2.44 µg m^−1^ L against *S. epidermidis* (9.8 µg mL^−1^ cefuroxime).	[92]
2-(*sec*-Butylthio)-3-((3-(trifluoromethyl)phenyl)amino)naphthalene-1,4-dione	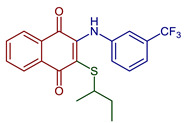 **50**	Antibacterial	MIC of 4.88 µgmL^−1^ against *S. epidermidis* (9.8 µgmL^−1^ cefuroxime).	[92]
*N*-(4-((3-Chloro-1,4-dioxo-1,4-dihydronaphthalen-2-yl)amino)phenyl)-R-benzenesulfonamide derivatives	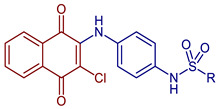 **51**, R= 4-methylphenyl **52**, R = 4-nitrophenyl	Antiviral	Anti-Chikungunya virus (CHIKV) activity, **51**, CC_50_ of 281 ± 2.5 µM and 99 ± 4.3% inhibition CHIKV replication; **52**, CC_50_ of 540 ± 3.7 µM and 98 ± 3.5% inhibition CHIKV replication.	[42]
2-Chloro-3-((3-(2-methylpiperidin-1-yl)propyl)amino)naphthalene-1,4-dione	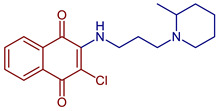 **53**	Anticancer	IC_50_ of 12.82 µM for HeLa cell.	[93]
2-Chloro-3-(phenyl(4-(phenylamino)phenyl)amino)naphthalene-1,4-dione	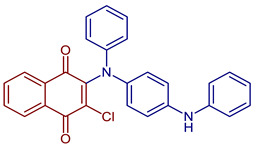 **54**	Anticancer	IC_50_ of 16.71 µM for HeLa cell.	[93]
2-Chloro-3-(methylphenylamino)naphthalene-1,4-dione	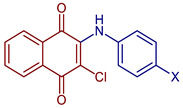 **55**, X=CH_3_	Anticancer	In vitro IC_50_ (μg mL^−1^) of 4.30± 0.46 against MOLT-3 cell line and 10.68 ± 1.89 against MDA-MB231 cell line. Doxorubicin and etoposide were used as reference drugs. Compound **55**, with an IC_50_ = 3.96 nM, could occupy the ATP-binding pocket of the target EGFR protein, similar to the pharmaceutical compound erlotinib EGFR inhibitor (IC_50_ = 16.17 nM).	[63]
2-Chloro-3-(cyanophenylamino)naphthalene-1,4-dione	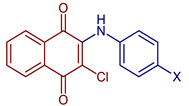 **56**, X=CN	Anticancer	IC_50_ (μg mL^−1^) of 1.75 ± 0.20 for MOLT-3 cell line. Doxorubicin and etoposide were used as reference drugs.	[63]
2-Chloro-3-(hydroxyphenylamino)naphthalene-1,4-dione	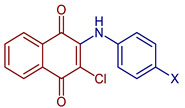 **57**, X=OH	Anticancer	IC_50_ (μg mL^−1^) of 8.21 ± 0.33 for HuCCA-1 cell line, and MDA-MB-231 cells. Doxorubicin and etoposide were used as reference drugs.	[63]
2-((4-(4-((1,4-Dioxo-1,4-dihydronaphthalen-2-yl)amino)piperidine-1-carbonyl)-3-methylphenyl)amino)-2-oxoethyl 4-methylbenzenesulfonate	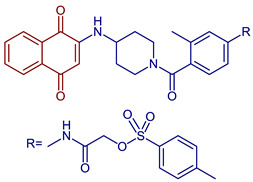 **58**	Anticancer	Compound **58** inhibits in vitro clone formation, induces apoptosis, inhibits cell migration and the arrest cell cycle, and blocks the STAT3 signaling pathway of gastric cancer cell MGC803 at IC_50_ = 0.57 µM. **58**, may be a promising STAT3 inhibitor for further developing potential anti-gastric cancer candidates.	[94]
2-Chloro-3-((2,4-dimethoxyphenyl)amino)-5-nitronaphthalene-1,4-dione	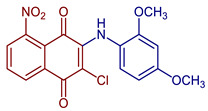 **59**	Catalase inhibitors related to several diseases	**59**, showed the strongest catalase enzyme inhibitory activity and highest antioxidant capacity with a 1.80 ± 0.06 CUPRAC-TEAC coefficient.	[68]
**Biological evaluations of NQs-amino acids**				
**Name**	**Chemical Structure**	**Potential** **Application**	**Evidence from Preclinical Assays**	**Ref**
2-((1,4-Dioxo-1,4-dihydronaphthalen-2-yl)amino)acetic acid	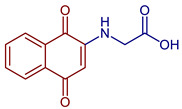 **60**	Antibacterial	In vitro antibacterial analysis showed MIC (µg mL^−1^) of 7.8 against *S. aureus* ATCC 25923, 31.2 against *E. coli* ATCC 25922, *E. faecalis* ATCC 29212, and *P. aeruginosa* ATCC 27853. **72** presented high gastrointestinal absorption and good characteristics for oral bioavailability.	[31]
		Anticancer	Inhibited ~80% of proliferation in SiHa cells and nearly 90% in MCF-7 cells.	[43]
2-((3-Chloro-1,4-dioxo-1,4-dihydronaphthalen-2-yl)amino)acetic acid	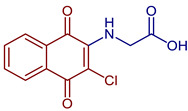 **61**	Anticancer	Compound **61** showed proliferation inhibition of 90% in MCF-7 cells.	[43]
2-((1,4-Dioxo-1,4-dihydronaphthalen-2-yl)amino)-3-phenylpropanoic acid	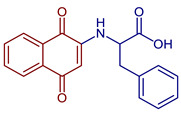 **62**	Antibacterial	In vitro compound **62** showed MIC of 24.7 µg mL^−1^ against *S. aureus* ATCC 25923, *E. coli* ATCC 25922, *E. faecalis* ATCC 29212, and *P. aeruginosa* ATCC 27853. Isolated clinical strains showed MICs of 49.7 µg mL^−1^ against *S. aureus* and 24.7 µg mL^−1^ against *E. coli* by 62*.	[31]
2-((3-Chloro-1,4-dioxo-1,4-dihydronaphthalen-2-yl)amino)-3-phenylpropanoic acid	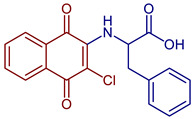 **63**	Antibacterial	In vitro antibacterial analysis showed MIC of 24.7 µg mL^−1^ against *S. aureus* ATCC 25923, *E. coli* ATCC 25922, *E. faecalis* ATCC 29212, and *P. aeruginosa* ATCC 27853. Isolated clinical strains showed MICs of 49.7 µg mL^−1^ against *S. aureus* and 24.7 µg mL^−1^ against *E. coli* by 63*.	[31]
2-((3-Chloro-1,4-dioxo-1,4-dihydronaphthalen-2-yl)amino)-3-(4-hydroxyphenyl)propanoic acid	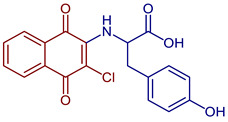 **64**	Anticancer	In vitro potent proliferation inhibition in cervical tumorigenic cell lines, showing an IC_50_ (µM) of 6.83, 7.028, ~0.001577 for SiHa, CaLo, and C33-A line cells, respectively.	[44]
4-Amino-2-((1,4-dioxo-1,4-dihydronaphthalen-2-yl)amino)-4-oxobutanoic acid	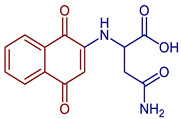 **65**	Antibacterial	In vitro antibacterial analysis showed MIC of 24.7 µg mL^−1^ against *S. aureus* ATCC 25923, *E. coli* ATCC 25922, *E. faecalis* ATCC 29212, and *P. aeruginosa* ATCC 27853. Isolated clinical strains showed MICs of 49.7 µg mL^−1^ against *S. aureus* and 24.7 µg mL^−1^ against *E. coli* by compound 65*.	[31]
		Anticancer	Inhibited ~80% of proliferation in SiHa cells.	[43]
3-Amino-2-((3-chloro-1,4-dioxo-1,4-dihydronaphthalen-2-yl)amino)-3-oxopropanoic acid	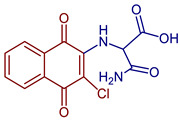 **66**	Anticancer	Showed proliferation inhibition ~85% in MCF-7 cells.	[43]
2-((3-Chloro-1,4-dioxo-1,4-dihydronaphthalen-2-yl)amino)-3-(1H-indol-2-yl)propanoic acid	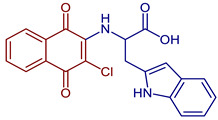 **67**	Anticancer	In vitro potent proliferation inhibition in cervical tumorigenic cell lines, showing an IC_50_ (µM) of 28.8, 25.20, and 21.36 for SiHa, CaLo, and C33-A cells, respectively.	[44]
*N*-Acetyl-*L*-cysteine naphthoquinone derivatives	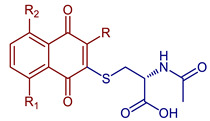 **68**, R=R_1_=R_2_=H **69**, R=CH_3_, R_1_=R_2_=H **70**, R=H, R_1_=OH, R_2_=H	Anticancer	Compounds **68**, **69**, and **70** showed potent cytostatic effects against HeLa, SH-SY5Y, SaOS2, and U2OS cancer cell lines with IC_50_ in the range of 0.50–1.81 µM.	[45]
**Biological evaluations of NQs-Mannich bases**				
**Name**	**Chemical Structure**	**Potential** **Application**	**Evidence from Preclinical Assays**	**Ref**
2-((Heptylamino)methyl)-3-hydroxynaphthalene-1,4-dione	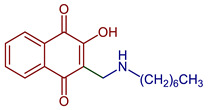 **71**	Antiparasitic	*T. gondii* (atovaquone-resistant) and *P. falciparum* (chloroquine-resistant) susceptible to compound **71**.	[95]
2-((Alkylamino)(pyridin-2-yl)methyl)-3-hydroxynaphthalene-1,4-dione	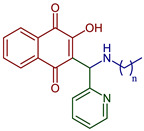 **72**, n=11 **73**, n=13 **74**, n=15	Antiparasitic	Compounds **72–74** showed sub-micromolar anti-trypanosomal activity against *T. brucei* via deformation of the microtubule cytoskeleton. Moreover, *N*-hexadecyl compound **74** was highly active against locally isolated *E. histolytica* parasite samples exceeding the activity of metronidazole.	[96]
		Anticancer	Compounds **72–74** exhibited strong and selective growth inhibitory activities in the low one-digit micromolar and sub-micromolar range against a panel of human cancer cell lines associated with ROS formation.	
2-Hydroxy-3-[(2-hydroxyphenyl)(hexylamino)methyl]naphthalene-1,4-dione hydrochloride 2-Hydroxy-3-[(2-hydroxyphenyl)(docecylamino)methyl]naphthalene-1,4-dione hydrochloride	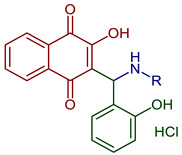 **75**, R=hexadecyl **76**, R=dodecyl	Antiparasitic	EC_50_ of 3.60 for **75** and 1.56 µM for **76** against *T. gondii* tachyzoites. Compounds displayed some selectivity for the *T. gondii* parasite compared to nonmalignant Vero cells with selectivity index (SI) values of 2.38 for **75** and 3.12 for **76**. Compound **75** exhibited EC_50_ of 10.2 and 3.62 µM for *L. major* promastigotes and amastigotes, respectively, and compound **76** showed EC_50_ of 5.57 and 4.16 µM for *L. major* promastigotes and amastigotes respectively (more efficacious that atovaquone). *T. b. brucei* was inhibited with an IC_50_ of 3.25 and 1.66 for **75** and **76**, respectively.	[97]
2-((Butylamino)methyl)-3-hydroxynaphthalene-1,4-dione	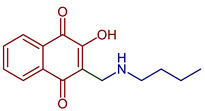 **77**	Antiparasitic	Antimalarial activity in vitro against *P. falciparum* with IC_50_ of 0.77 μg mL^−1^ (*P. falciparum* K1, multidrug-resistant strain).	[90]
2-(((4-Fluorophenyl)amino)(phenyl)methyl)-3-hydroxynaphthalene-1,4-dione	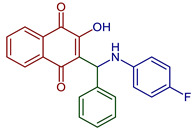 **78**	Antiparasitic	Compound **78** showed antimalarial activity with IC_50_ 0.423 and 1.492 µg mL^−1^ for Chloroquine(CQ) –sensitive (3D-7) and CQ–resistant (RKL-2) strains of *P. falciparum,* respectively.	[51]
2-Hydroxy-3-(((3-nitrophenyl)amino)(R-phenyl)methyl)naphthalene-1,4-dione derivatives	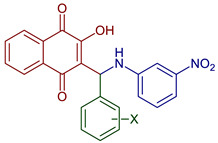 **79**, X=4NO_2_ **80**, X=2OH	Antiparasitic	Compound **79** showed antimalarial activity with IC_50_ 0.475 and 1.391 µg mL^−1^ for CQ–sensitive (3D-7) and CQ–resistant (RKL-2) strains of *P. falciparum,* respectively. Compound **80** exhibited IC_50_ 0.502 and 2.394 µg mL^−1^ for CQ–sensitive (3D-7) and CQ–resistant (RKL-2) strains of *P. falciparum,* respectively.	[51]
2-Hydroxy-3-(R-phenyl(pyrrolidin-1-yl)methyl)naphthalene-1,4-dione	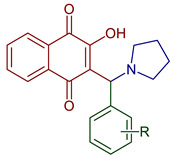 **81**, R=H **82**, R=2OH	Antiparasitic	Compound **81** showed antimalarial activity with IC_50_ 0.412 and 2.212 µg mL^−1^ for CQ–sensitive (3D-7) and CQ–resistant (RKL-2) strains of *P. falciparum,* respectively. Compound **82** exhibited IC_50_ 0.411 and 1.170 µg mL^−1^ for CQ–sensitive (3D-7) and CQ–resistant (RKL-2) strains of *P. falciparum,* respectively.	[51]
	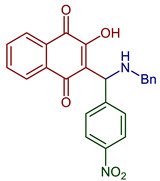 **83**	Antiviral	Antiviral action of **83** against BoHV5 CC_50_ of 1867 ± 8.3 µM and EC_50_ 3.8 ± 1.2 µM (Acyclovir: CC_50_ of 989 ± 2 µM and EC_50_ of 166 ± 2 µM).	[98]
2-((Butylamino)(2,4-dichlorophenyl)methyl)-3-hydroxynaphthalene-1,4-dione 2-((Benzylamino)(2,4-dichlorophenyl)methyl)-3-hydroxynaphthalene-1,4-dione	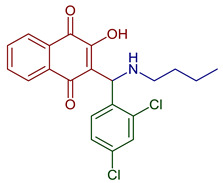 **84** 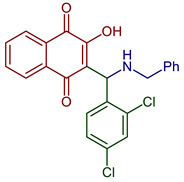 **85**	Antiviral	Compounds **84** and **85** affect the L-phase of the HSV-1 replicative cycle by gD protein expression inhibition. The nature of the substituent on the nitrogen atom, the conformation, and the LUMO distribution of benzyl portion versus *n*-butyl substituents modulates antiviral activity. Recently, antiviral activity with EC_50_ = 1.73 ± 0.08 µM for **84** and 0.56 ± 0.02 for **85** encapsulated in liposomes.	[99,100]
3-(4-(((3-Bromo-1,4-dioxo-1,4-dihydronaphthalen-2-yl)amino)methyl)-1*H*-1,2,3-triazol-1-yl)-2,2-dimethyl-2,3-dihydronaphtho[1,2-*b*]furan-4,5-dione	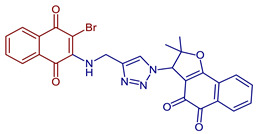 **86**	Antiviral	Compound **86** at 10 µM showed 100% protease (Mpro)-SARS-CoV-2 inhibition action with IC_50_ = 1.9 ± 0.06 µM.	[101]
2,3-Bis(phenylthio)naphthalene-1,4-dione	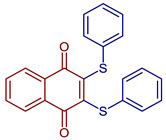 **87**	Antiviral	Compound **87** at 10 µM showed 100% protease (Mpro)-SARS-CoV-2 inhibition action with IC_50_ = 0.63 ± 0.04 µM.	[101]
2,3-Bis((4-methoxyphenyl)thio)-5-nitronaphthalene-1,4-dione	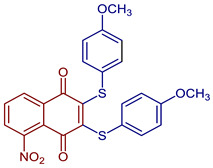 **88**	Antiviral, SARS-CoV-2, Mpro inhibitors	Compound **88** at 10 µM showed 100% protease (Mpro)-SARS-CoV-2 inhibition action with IC_50_ = 0.41 ± 0.02 µM.	[101]
*N*-(5-Nitro-1,4-dioxo-1,4-dihydronaphthalen-2-yl)acetamide	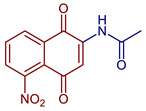 **89**	Antiviral, SARS-CoV-2, Mpro inhibitors	Compound **89** at 10 µM showed 100% protease (Mpro)-SARS-CoV-2 inhibition action with IC_50_ = 9 ± 1 µM.	[101]
Ferrocene 2-(amino(pyridin-2-yl)methyl)-3-hydroxynaphthalene-1,4-dione derivative	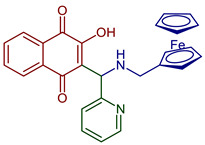 **90**	Anticancer	Antiproliferative effects in the androgen-receptor negative PC-3 prostate and Pgp expressing KB-V1/Vb1 cervix carcinoma cell lines at sub-micromolar concentration.	[102]
2-Hydroxy-3-((octylamino)(R-phenyl)methyl)naphthalene-1,4-dione	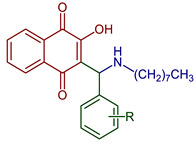 **91**, R: 2OH **92**, R: OH, 5Br	Anticancer	Compounds **91** and **92** were active with IC_50_ of 11.68 and 1.64 µM against the HepG2 line cell, respectively.	[52]
2-(2-Alkyl-3-oxo-2,3-dihydro-1*H*-isoindol-1-yl)-3-hydroxynaphthalene-1,4-dione derivatives	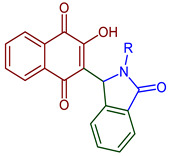 **93**, R=alkylamine	Anticancer	Isoindolinone derivatives enhanced cancer cell death and prevention of tumor growth by restoring serum SGOT and SGPT levels near to normal; docking studies revealed an association on promising liver cancer-associated Alpha-fetoprotein (AFP)].	[53]
Naphthoquinone polyphenols derivatives	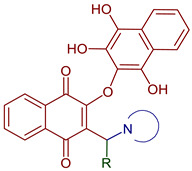 **94**, 16 examples	Anticancer	Several polyphenols were tested on four cancer cell lines (HCT116, PC3, HL60, and SNB19), in which the best results showed antiproliferative activity with IC_50_ of 25.83 to 47.95 μM. Additionally, the antioxidant activity was determined using the CRAC assay.	[103]
11-(5,6,7,8-Tetrahydronaphthalen-2-yl)-*1H*-R-benzo[*g*]cyclopenta[b]quinoline-1,5,10(4*H*,11*H*)-trione	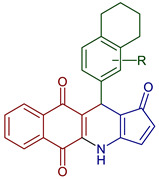 **95**: R=H, **96**: 3Br, **97**: 3NO_2_, **98**: 3OMe	Anticancer	Podophyllotoxin-naphthoquinone derivatives. Compounds **95**, **96**, **97**, and **98** displayed highly potent inhibitory activities with IC_50_ < 40 nM against HepG2 and SK-Lu-1 cell lines and showed lower toxicity for the non-cancerous Hek-293 cell line.	[104]
3,3’-Methylene)Bis-2-hydroxy-1,4-naphthoquinones derivatives	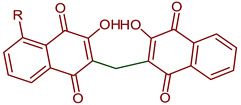 **99**: R=H **100**: R=OH	Anticancer	Compounds **99** and **100** induced cytotoxicity against DU145 and PC3 cells. Promoted cell cycle arrest in G1/S and G2/M phases, increased Sub-G1 peak and inhibited cell viability.	[105]

MIC: minimum inhibitory concentration; CC50: 50% cytotoxic concentration; IC_50_: 50% inhibitory concentration; EC_50_: 50% effective concentration. TEAC: trolox equivalent antioxidant capacity coefficient; *S. aureus*: *Staphylococcus aureus*; *M. luteum*: *Mycobacterium luteum*; *S. epidermidis*: *Streptococcus epidermidis*; *E. faecalis*: *Enterococcus faecalis*; *P. aeruginosa*: *Pseudomonas aeruginosa*; *T. gondii: Toxoplasma gondii; P. falciparum: Plasmodium falciparum; T. b. brucei: Trypanosoma brucei brucei; E. histolytica: Entamoeba histolytica.* BoHV5: bovine herpes virus type 5; HSV-1: herpes simplex viruses. MOLT-3: lymphoblastic leukemia cell line; MDA-MB-231 hormone-independent breast cancer cell line; HuCCA-1 cholangiocarcinoma cancer cell line; SiHa: cervical tumor line cell line; MCF-7: breast cancer cells; CaLo: cervical tumor line; C33-A: cervical tumor line; SH-SY5Y SaOS2, U2OS cancer cell; Vero (African green monkey kidney epithelial); A549 lung carcinoma cell line, HeLa: human cervical carcinoma; HepG2 hepatocellular carcinoma cell line, T47D hormone-dependent breast cancer cell line; MRC-5 normal embryonic lung cell line; PCa: prostate cancer cell lines DU145 and PC3. EGFR: epidermal growth factor receptor; STAT3: signal transducer and activator of transcription 3. * In ADME properties and Osiris analysis, presented high gastrointestinal absorption and good characteristics for oral bioavailability.

## Data Availability

Data sharing not applicable.
